# Supplementary material for: Observation of replica symmetry breaking in disordered nonlinear wave propagation
Source: Nat Commun. 2017 Nov 15;8:1501. doi: 10.1038/s41467-017-01612-2 (PMC5688108; doi:10.1038/s41467-017-01612-2)
Supplement: Supplementary file 1 — Supplementary Information [file 41467_2017_1612_MOESM1_ESM.pdf]

Supplementary Note 1

Theoretical framework

In general, nonlinear optical wave propagation in paraxial conditions is described by the generalized nonlinear Schrödinger equation (GNLSE) for the optical field evelope $\psi(x, y, z)$,

$$i\frac{\partial\psi}{\partial z} + \frac{1}{2k}\nabla^2\psi + U(x,y,z)\psi + \frac{k\delta n(\psi)}{n}\psi = 0, \tag{1}$$

where $U(x, y, z)$ can be an ordered or disordered optical potential and $\delta n(\psi) = \delta n(|\psi|^2) = \delta n(|\psi(x, y, z)|^2)$ accounts for the nonlinear response of the supporting medium. In our case, the focusing photorefractive nonlinearity in centrosymmetric media at the leading order reads as $\delta n(\psi) = \Delta n_0/(1 + |\psi|^2)^2$, where Δn_0 includes the extremely large response of the medium [1–3]. Experiments are performed in the so-called saturated regime, i.e., the input peak-amplitude of the wave ψ_0 satisfies $|\psi_0|^2 > 1$ [4]. In general, paraxiality of the waves allows us to consider the optical potential as slowly varying along the propagation direction respect to the transverse coordinates, that is, $U(x, y, z) = V_{\mathrm{r}}(x)+V_{\mathrm{g}}(y)+V_{\mathrm{r}}(z)$, where $V_{\mathrm{g}}(y)$ represents the slab waveguide profile, $V_{\mathrm{r}}(x)$ and $V_{\mathrm{r}}(z)$ are, respectively, the disordered component along x and z. The disordered potential $V_{\mathrm{r}}(z)$ can be included into a noise term, whereas the slab waveguide allows us to consider the one-dimensional counterpart of Supplementary Equation (1). Transverse modes thus satisfy the z-independent linear Schrödinger equation

$$\frac{1}{2k}\frac{\partial^2\psi_n(x)}{\partial x^2} + V_{\mathrm{r}}(x)\psi_n(x) = \beta_n\phi_n(x), \tag{2}$$

where the eigenvalues β_n are propagating wave vectors. Interaction between modes can be treated variationally by letting

$$\psi(x, z) = \sum_n a_n(z)\psi_n(x)e^{i\beta_n z}. \tag{3}$$

Mode amplitudes thus evolve according to

$$i\dot{a}_n(z) = i\sum_j G^{(2)}_{jn} a_j\beta_j e^{i(\beta_j-\beta_n)z} + \sum_{jlk} G^{(4)}_{jlkn} a^*_j a_k a_l e^{i(\beta_j+\beta_l-\beta_k-\beta_m)z} \tag{4}$$

where $G^{(2)}_{jn}$ and $G^{(4)}_{jlkn}$ are, respectively, the linear and nonlinear mode-overlap coefficients [5]. It should be noted that the global sign of $G^{(4)}_{jlkn}$ depends on the focusing/defocusing character of the nonlinearity and does not alter the RSB scenario [5]. This nonlinear coupling is a key ingredient for the breaking of replica symmetry in optical propagation since optical modes generally are at most weakly interacting in the linear regime (due to orthogonality or weakness of the linear perturbation). Considering the corresponding Langevin dynamical equations

$$\dot{a}_n(z) = -\frac{\partial\mathcal{H}}{\partial a^*_n(z)} + \eta_n(z), \tag{5}$$

where $\eta_n(z)$ is the noise term that takes into account linear and nonlinear random fluctuations of the medium along the propagation, Supplementary Equation (4) implies a spin-glass-like Hamiltonian of the form

$$\mathcal{H} = -\frac{1}{2!}\sum_{jk} G^{(2)}_{jk} a_j a^*_k - \frac{1}{4!}\sum_{jklm} G^{(4)}_{jklm} a_j a^*_k a_l a^*_m. \tag{6}$$

Here the complex amplitudes a_j are subject to the global power constraint $\mathcal{E} = \sum_j |a_j|^2$ =const and nonlinear interaction runs over modes satisfying the resonant condition

$$|\beta_j + \beta_l - \beta_k - \beta_m| < \gamma, \tag{7}$$

being γ the typical linewidth of the modes. This requirement for mode interaction is easily satisfied in our case due to the paraxial regime and the slab waveguide, which ensure that several modes are densely packed.

As detailed in [5–7], a mean-field approach to Supplementary Equation (6) with fixed disordered couplings gives a phase diagram as a function of the degree of disorder and nonlinearity that includes a phase transition from a paramagnetic-like phase to a glassy state. Specifically, in Supplementary Figure 1 a phase diagram as expected for

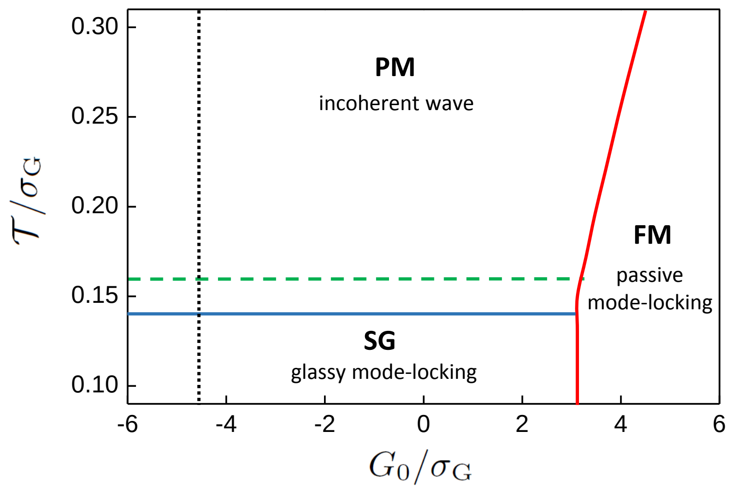

Supplementary Figure 1. Phase diagram for nonlinear disordered propagation. $G_0/\sigma_{\rm G}$ is the inverse degree of disorder whereas $\mathcal{T}/\sigma_{\rm G}$ quantifies the inverse nonlinearity strength (arbitrary units). Three phases separated by thermodynamic transitions (solid lines) are indicated: paramagnetic-like (PM), ferromagnetic-like (FM) and spin-glass-like (SG). The black dotted line indicates the region in which nonlinear dynamics has been experimentally investigated. A transition from PM to SG as nonlinearity increases is predicted and experimentally observed at fixed degree of disorder.

nonlinear wave propagation is reported [5], which, at variance with the active cavity cases of random lasers [6, 7], includes both positive (defocusing) and negative (focusing) average couplings. The degree of disorder is expressed through the mean coupling strength $G_0 = G_0^{(2)} + G_0^{(2)}$ and the standard deviation of the distribution of the coupling coefficients $\sigma_{\rm G} = \sigma_{\rm G}^{(2)} + \sigma_{\rm G}^{(4)}$. The limit $G_0/\sigma_{\rm G} \to 0$ corresponds to the completely disordered case. Nonlinearity is expressed in terms of the environmental temperature $\mathcal{T}$, which, in nonlinear optical propagation, can be related to the nonlinear coefficient δn. The dotted line in Supplementary Figure 1 indicates the region of the phase space in which evolution of the system takes place in our experiments. A transition to a glassy phase in which modes are randomly locked occurs as the nonlinearity exceeds a threshold value, even for small degrees of disorder ($G_0/\sigma_{\rm G} < 0$ and $|G_0/\sigma_{\rm G}| \gg 1$). This transition is characterized by a replica symmetry breaking (RSB) in the overlaps $Q_{ab} = \frac{1}{\mathcal{E}} \sum_j a_j^a (a_j^b)^*$: the overlap distribution $P(Q)$ continuously passes from a distribution peaked around $Q = 0$ to one having its maximum at $|Q| = 1$. Generally, $Q = 0$ indicates random correlations between different realizations of the dynamics, whereas $Q = 1$ implies totally correlated or anticorrelated properties depending on the specific replica pair. The nature of this transition can be analogous to that leading to random lasing in open disorder cavities [7], which is a mixed process continuous in the order parameter including one-step plus full replica symmetry breaking (1RSB + FRSB).

It is important to stress how the RSB property does not strictly depends on the specific nature of the involved modes a_j. The key point is that, whatever the modes identified, they should be all randomly coupled by the interaction. In our experiments, spontaneous inhomogeneities of the medium constitute the disordered weak optical potential $V_r(x)$; its modes are weakly localized and, to a first approximation, they can be considered as plane waves. However, mode eigenvalues are propagating wave vectors and cannot be experimentally accessed. To investigate nonlinear mode dynamics the entire spatial intensity distribution of the field has to be considered. Since we measure the output intensity distribution $I(x) = |\psi(x)|^2$, it is useful to express the field in the positional basis as

$$\psi(x,z) = \sum_j A(z,x_j)\psi_j(x - x_j), \tag{8}$$

where $\psi(x_j - x_i) = \delta_{ij}$ are cardinal functions [8] and the amplitudes $A(z,x_j) = A_j(z)$ now include all the propagation dependence. These spatial amplitudes are linear combinations of the original mode amplitudes

$$A_j(z) = \sum_n a_n(z)\psi_n(x_j) = \psi(x_j) \tag{9}$$

and thus present the same breaking of the replica symmetry. In fact, the coupled mode equation reads as

$$\frac{dA_i}{dz} = \sum_j \mathcal{K}^{(2)}(x_i - x_j)A_j(z) + \sum_{jkl} \mathcal{K}^{(4)}(x_i - x_j, x_i - x_k, x_i - x_l)A_j(z)^* A_k(z)A_l(z). \tag{10}$$

Supplementary Equation (10) leads to an Hamiltonian spin-glass model analogous to Supplementary Equation (6) in which the coupling coefficients $\mathcal{K}^{(2)}, \mathcal{K}^{(4)}$ are now dependent on the spatial distance between transverse points of the field rather than on the mode index. The overlap parameter whose probability distribution signals replica symmetry breaking is thus $Q_{ab} = \frac{1}{\mathcal{E}} \sum_j A_j^a(z)(A_j^b(z))^*$. This quantity cannot be directly accessed in experiments since $A_j(z)$ is the complex field amplitude. At a fixed z, we thus consider the single-shot autocorrelation function $g_a(x) = \sum_{x'} I_a(x')I_a(x'+x)$. Mode interaction and locking generally result into typical features of the autocorrelation function since phase correlations between different modes contribute as constructive and destructive interference effects at distance x. In this case, shot-to-shot fluctuations are defined as $\Delta_a(x) = g_a(x) - \bar{g}(x)$, where $\bar{g}(x)$ is the autocorrelation averaged over all the realizations. The experimentally accessible overlap we measure is then

$$q_{ab} = \frac{\sum_x^R \Delta_a(x)\Delta_b(x)}{\sqrt{\sum_x^R (\Delta_a(x))^2}\sqrt{\sum_x^R (\Delta_b(x))^2}}, \tag{11}$$

where R is the maximum distance at which spatial correlations are calculated. Neglecting variations of the normalization factors $\sqrt{\sum_x^R (\Delta_a(x))^2}$ with respect to shot-to-shot fluctuations $\Delta_a(x)$, the overlap parameter can be written in terms of the spatial mode amplitudes in Eqs. (9)-(10) as

$$\begin{aligned} q_{ab} =& \sum_x^R \left\langle \sum_{x'} |A_a(x')|^2 |A_a(x'+x)|^2 |A_b(x')|^2 |A_b(x'+x)|^2 \right\rangle - \\ & \sum_x^R \left\langle \sum_{x'} |A_a(x')|^2 |A_a(x'+x)|^2 \right\rangle \left\langle \sum_{x'} |A_b(x')|^2 |A_b(x'+x)|^2 \right\rangle \end{aligned} \tag{12}$$

where the average can be carried out using the replica method. In close analogy with Q_{ab}, a replica symmetry breaking transition is expected to occur for the q_{ab} distribution [7]. Our experimental results directly point out this glass transition, in spite of the averaging over different realizations of the disorder.

We note that, in principle, a direct quantity to characterize the replicas would be their bare spatial intensity distributions. However, since the intensity strongly varies on a small scale of the order of few times the experimental resolution ($\approx 0.3\mu$m), in our case this analysis is unstable with respect to the method we have used to obtain each realization. In fact, for each shot the experiment is entirely repeated and, practically, the system cannot be maintained in the same position on the experimental resolution. Even the thermal expansion of the crystal associated to the heating process would be enough to lose this fine alignment. This fact introduces an arbitrary spatial shift of the output intensity distribution from shot to shot, which acts as an artificial randomization of the spatial coordinate and inevitably alters the overlap evaluation through the local intensity distribution. On the other hand, the intensity autocorrelation is not affected by this experimental limitation as it is an averaged quantity depending only on the relative spatial coordinate.

Supplementary Note 2

Experimental details

In detecting the breaking of the replica symmetry for increasing interaction strength the time-dependent property of the photorefractive nonlinearity has been experimentally used. In this Supplementary Note we provide a direct connection between the exposure time and the light-induced nonlinear index change. As the light beam impinges on the biased photorefractive crystal it starts to generate an illumination-dependent variation of the index of refraction by means of carriers excitation and their spatial redistribution [4]. This process occurs on a slow time scale, typically seconds for peak intensities of few kW cm^{-2} [9]. Owing to the propagating wave field being strongly inhomogeneous in space, the light-induced index change $\delta n(\psi)$ strictly depends on the specific spatial point, so that only an average time-dependence of the nonlinearity is meaningful. A direct measurement of this spatially-averaged index variation $\Delta n(t) =< \delta n(t) >$ while the nonlinear dynamics is underway is not at all trivial since it would require the whole complex amplitude of the field. Experimental results obtained in similar photorefractive KTN crystals close to their ferroelectric phase-transition have verified that the average index change grows and saturates according to $\Delta n(t) = \Delta n_0(1 - \exp(-t/\tau))$ [10, 11]. This time dependence is well defined through the time scale τ once the input beam intensity, applied voltage and temperature have been fixed. Following these studies, we have numerically evaluated $\Delta n(t)$ considering the values of the parameters used in our experiments. In Supplementary Figure 2 the nonlinearity strength as a function of time has been reported for the cases shown in Fig. 2(a) and Fig. 2(b),

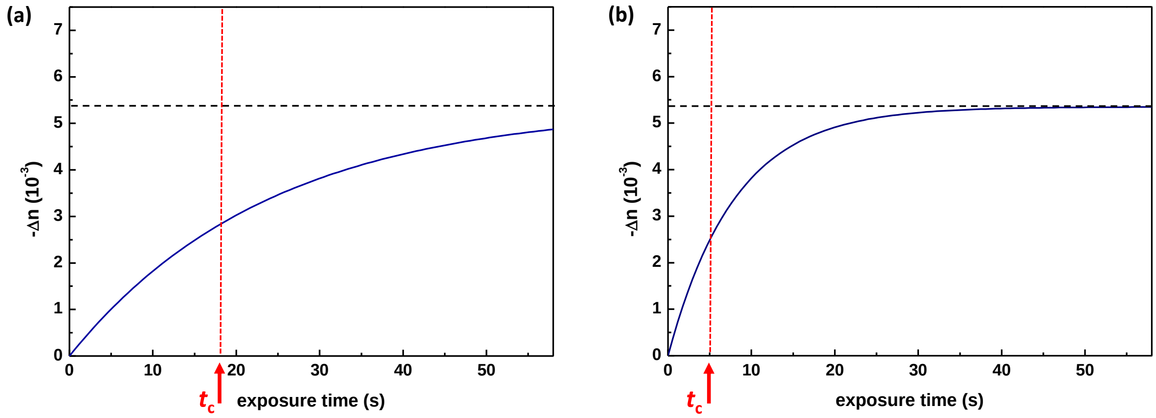

Supplementary Figure 2. Time-dependence of the nonlinearity strength. Average index change $\Delta n(t)$ numerically evaluated for the dynamics reported in (a) Fig. 2(a) for $P = 0.2$ mW and (b) Fig. 2(b) for $P = 5$ mW. Black dashed lines indicate the intensity-independent saturation value Δn_0. Red dotted lines at t_c signal the optical glass transitions as observed in Fig. 2.

respectively. $\Delta n(t)$ grows with the exposure time up to a saturation value and this behavior occurs as fast as the input intensity. Consistently, compatible values of the effective nonlinearity for the glass transitions observed at t_c are found under different input powers (Supplementary Figure 2). For long times the saturation value is reached, that is, the nonlinearity as well as the light distribution reach a stationary state and no further changes take place over time at fixed propagation distance.

Supplementary References

[1] DelRe, E., Spinozzi, E., Agranat, A.J. & Conti, C. Scale-free optics and diffractionless waves in nanodisordered ferroelectrics, *Nat. Photonics* **5**, 39-42 (2011).

[2] Pierangeli, D. *et al.* Photorefractive light needles in glassy nanodisordered KNTN, *Opt. Lett.* **39**, 1657-1660 (2014).

[3] Pierangeli, D., Di Mei, F., Conti, C., Agranat, A.J. & DelRe, E. Spatial Rogue Waves in Photorefractive Ferroelectrics, *Phys. Rev. Lett.* **115**, 093901 (2015).

[4] DelRe, E., Crosignani, B. & Di Porto, P. Photorefractive Solitons and Their Underlying Nonlocal Physics, *Progress in Optics* **53**, 153-200 (2009).

[5] Conti, C. & Leuzzi, L. Complexity of waves in nonlinear disordered media, *Phys. Rev. B* **83**, 134204 (2011).

[6] Antenucci, F., Conti, C., Crisanti, A. & Leuzzi, L. General Phase Diagram of Multimodal Ordered and Disordered Lasers in Closed and Open Cavities, *Phys. Rev. Lett.* **114**, 043901 (2015).

[7] Antenucci, F., Crisanti, A. & Leuzzi, L. The glassy random laser: replica symmetry breaking in the intensity fluctuations of emission spectra, *Sci. Rep.* **5**, 16792 (2015).

[8] Boyd, J.P. *Chebyshev and Fourier Spectral Methods*, Second Edition (Dover, New York 2001).

[9] DelRe, E., Ciattoni, A. & Palange, E. Role of charge saturation in photorefractive dynamics of micron-sized beams and departure from soliton behavior, *Phys. Rev. E* **73**, 017601 (2006).

[10] Lu, Q., Han, J., Dai, H., Ge, B. & Zhao, S. Visualization of spatialtemporal evolution of light-induced refractive index in Mn: Fe: KTN Co-doped crystal based on digital holographic interferometry, *IEEE Photonics J.* **7**, 2600711 (2015).

[11] Lu, Q., Li, B., Dai, H., Ge, B. & Han, J. In situ observation and monitoring of the dynamic behavior of field-induced photorefractive grating formation process with digital holographic microscopy, *Opt. Mat. Express* **6**, 2991 (2016).
